# Variation in hemolytic activity of *Brachyspira hyodysenteriae* strains from pigs

**DOI:** 10.1186/s13567-016-0353-x

**Published:** 2016-06-23

**Authors:** Maxime Mahu, Nele De Pauw, Lien Vande Maele, Marc Verlinden, Filip Boyen, Richard Ducatelle, Freddy Haesebrouck, An Martel, Frank Pasmans

**Affiliations:** Department of Pathology, Bacteriology and Avian Diseases, Faculty of Veterinary Medicine, Ghent University, Salisburylaan 133, B-9820 Merelbeke, Belgium; Technology and Food Science Unit, Institute for Agricultural and Fisheries Research (ILVO), Brusselsesteenweg 370, B-9090 Melle, Belgium

## Abstract

*Brachyspira hyodysenteriae* is the primary cause of swine dysentery, which is responsible for major economic losses to the pig industry worldwide. The hemolytic activity of 10 *B. hyodysenteriae* strains isolated from stools of pigs with mild to mucohemorrhagic diarrhea was compared and seven hemolysis associated genes were sequenced. Hemolysis induced by these strains varied from strong to near absent. One weakly hemolytic *B. hyodysenteriae* strain showed sequence changes in five hemolysis associated genes (*tlyA, tlyB, hemolysin III*, *hemolysin activation protein* and *hemolysin III channel protein*) resulting in amino acid substitutions. The occurrence of weakly hemolytic strains identifiable as *B. hyodysenteriae* should be taken into account in swine dysentery diagnostics. The presence of these strains may affect herd dysentery status, with great impact on a farms trading opportunities.

## Introduction

Swine dysentery (SD) is caused by the anaerobic spirochete *Brachyspira hyodysenteriae* and is an important intestinal disease in swine rearing countries. Clinical signs typically consist of mucohemorrhagic diarrhea. The economic losses on farm level can be substantial due to mortality, diminished growth rates, deterioration of feed conversion and costs of medical treatment [1]. The occurrence of *B. hyodysenteriae* in a herd may affect the trading of pigs with economic consequences, even in the absence of overt clinical signs. Diagnostics of herds for the presence of *B. hyodysenteriae* is usually done by examining pooled fecal samples for the presence of this micro-organism by microbial culture and/or PCR tests [2].

Besides *B. hyodysenteriae,* other *Brachyspira* species of varying virulence have been described in pigs. There has been an interest in putative virulence factors to help explain the differential pathogenic potential of these different *Brachyspira* spp. Possible virulence factors include motility, chemotactic capacities, lipopolysaccharide, hemolysin(s) and enzymes such as NADH oxidase [1, 3].

The pronounced hemolysis of *B.hyodysenteriae* that is displayed by growth on blood agar plates has been considered a hallmark of its pathogenicity [4, 5]. Apart from *B.pilosicoli*, weakly hemolytic porcine *Brachyspira* spp. such as *B.murdochii* and *B.intermedia* are indeed regarded mildly or non-pathogenic. Although they have been isolated from clinical cases of diarrhea [6], their pathogenic potential is less clear-cut since experimental infections require large numbers of spirochetes and clinical symptoms are either mild or absent [7]. On the other hand, virulence of the recently described strongly hemolytic *B.suanatina* and “*B.hampsonii*” is considered to be similar to that of *B. hyodysenteriae* [8–13].

Several reports describe the purification of hemolysin produced by *B.hyodysenteriae* [4, 5, 14]. Using purified hemolysin in an ileal-colonic loop model, microscopic lesions similar to those seen in natural cases of swine dysentery have been reproduced [15]. Four hemolysis associated genes have been defined: *tlyA, tlyB*, *tlyC* and *hlyA* [16–18]. The protein encoded by *tlyA,* hemolysin A, shows homology with pore forming hemolysins of several bacteria such as *Mycobacterium tuberculosis* [19, 20], and *Treponema denticola* [21]. These homologues and Hemolysin A also encompass a conserved domain which is predicted to function as a rRNA methyltransferase [21]. *TlyA* negative *B.* *hyodysenteriae* mutants are less hemolytic and induce less severe lesions in mice and pigs compared to their wildtype [22, 23]. The *TlyB* gene encodes a Clp protease, and *tlyC* encodes hemolysin C. Both recombinant proteins were proven to show hemolytic and cytotoxic activity in vitro [17]. Bellgard et al. [24] describe that, in order to display a hemolytic phenotype, *B.* *hyodysenteriae* could need an acyl carrier protein (ACP) for acylation of toxins. Such an ACP is encoded for by *hlyA. ThefabF* and *fabG* genes encode an ACP-reductase and synthase that presumably play a role in the lipidation of the HlyA protein [25]. Even though some weakly hemolytic *Brachyspira* spp. strains also contain the *hlyA* gene, it is probably not functional due to incorrect localization between the *fab* genes [26].

In addition to previously described hemolysis related genes *tlyA*, *tlyB*, *tlyC* and *hlyA*-ACP Bellgard et al. [24] found three possibly important additional genes when the whole genome sequence of reference strain WA1 was described: *hemolysin III*, *hemolysin activation protein* and *hemolysin III channel protein* genes. Hemolysin III (BHWA1_RS02195) [24], encompasses a conserved domain yqfA, a predicted channel-forming protein of the hemolysin III family. Homologues of hemolysin III are found in several bacteria such as *Bacillus cereus* [27]. The hemolysin III related channel protein (BHWA1_RS09085) [24], has a conserved domain composing an integral membrane protein. The hemolysin activation protein (BHWA1_RS02885) [24], shares conserved domains with hemolysin C.

We previously mentioned the existence of *B.hyodysenteriae* strains with an aberrant hemolytic phenotype [28]. In 1982, Lysons et al. [29] isolated three strains of *B.hyodysenteriae* that were reported to appear slightly less hemolytic on blood-containing agar plate than virulent strains of *B.hyodysenteriae*, though considerably more hemolytic than avirulent *B.innocens*. Disease signs could not be induced using two of these strains in an in vivo experiment, even when animals were colonized by the strain. Recently, Hampson et al. [30] described the existence of weakly hemolytic *B.hyodysenteriae* strains in Australia as well. The current study aims to quantify the hemolytic capacity of a selection of *B.hyodysenteriae* strains and to identify the underlying molecular differences.

## Materials and methods

### *Brachyspira* isolate collection and selection

A collection of isolates of different *Brachyspira* species was composed during a 6 month period (Oct 2011–March 2012) at our facilities. *B.hyodysenteriae* isolates were collected by the participation of swine veterinarians who were asked to share fresh fecal samples, if *B.hyodysenteriae* infection was suspected on a farm based on clinical symptoms. Furthermore two diagnostic laboratories (Animal Healthcare Flanders, Drongen, Belgium and Mediclab, Aalst, Belgium) donated isolates of *B.* *hyodysenteriae* and other porcine associated *Brachyspira* species that they had collected during the past 2 years (2010–2011).

Participating swine veterinarians collected two or three pooled fecal samples (3 pigs per pooled sample) on each farm which were cultured within 24 h after sampling on selective plates consisting of Trypticase Soy Agar (TSA) (Sigma-Aldrich, St. Louis, MO, USA) supplemented with 5% sheep blood (E&O Laboratories, Bonnybridge, UK), 1% yeast extract (Becton–Dickinson, Franklin Lakes, NJ, USA), 25 µg/mL vancomycin, 400 µg/mL spectinomycin and 25 µg/mL colistin (all antimicrobial compounds from Sigma-Aldrich). Plates were anaerobically incubated at 38 °C. Isolates were purified by three to five subcultures on Trypticase Soy Agar (TSA) plates supplemented with 5% sheep blood and 1% yeast extract [31] and eventually stored at −70 °C in 300 µL of a medium consisting of 75 mL horse serum (Thermo Fisher Scientific, Carlsbad CA, USA) and 25 mL Brain Heart Infusion broth (Bio-Rad, Hercules CA, USA) supplemented with 10% (w/v) glucose (Merck, Darmstadt, Germany) until further use. Isolates donated by diagnostic laboratories were delivered on agar plates. All isolates were subcultured once after which they were also stored at −70 °C. All donated isolates were accompanied by a brief description of clinical symptoms on the farm of origin.

On all collected isolates phenotypic characterization and species-specific PCR’s were performed. Phenotypic characterization was performed on pure 4-day old cultures and was based on beta-hemolysis, indole production, hippurate hydrolysis and the presence or absence of α-galactosidase and β-glucosidase [32, 33]. Indole production was determined using a spot-indole test (Remel BactiDrop, Dartford, UK). For the other biochemical characteristics, commercial discs were used according to the manufacturer’s instructions (Rosco Diatabs, Taastrup, Denmark). Type strains of *B.hyodysenteriae* (ATCC 27164)*, B.pilosicoli* (ATCC 51139) and *B.innocens* (ATCC 29796) were included to provide positive controls for all the phenotypic characteristics that were examined.

Three *B.hyodysenteriae*-specific PCRs were performed, based on the following genes: *tlyA* [34], 23S rRNA [35] and *nox* [36]. Species-specific PCR for the other species were based on *nox* [37] and 23S rRNA [35] for *B. intermedia*, 16S rRNA [36] for *B. pilosicoli* and *nox* [38] for *B.murdochii/B.innocens.*

Out of the complete collection of *B.hyodysenteriae* isolates, 8 were selected at random to be evaluated in an in vitro assay for hemolytic capacity, MLST profiling, and sequence analysis of 16S rRNA, the NADH oxidase gene, and hemolysis associated genes. Two more isolates were specifically selected for the same assays, since they showed an aberrant phenotype when grown on blood containing agar plates: M2 showed only moderate hemolysis and isolate D28 showed weak hemolysis. Strain B204 (ATCC 31212) was included as a positive control. These 10 selected isolates, the year of isolation, and the clinical symptoms on the farm of origin are given in Table 1. All isolates originated from different, non-related farms, except isolates M1 and M2, which originated from the same farm. M1 was isolated from fecal samples of finisher pigs, M2 was isolated from fecal samples of growing pigs. Both age groups suffered from mucohaemorrhagic diarrhea.

**Table 1:** **Clinical signs on the farm of origin, phenotypic characteristics, MLST profile and sequence type, 16S rRNA and** ***nox*** **sequence lengths and accession numbers**

Strain ID^a^	Year of isolation	Clinical signs on the farm of origin^b^	Hemolysis on agar plate^c^	Enzymatic profile^d^	MLST profile (sequence type)^e^	*Nox* sequence accession number and sequence length (bp)	16S rRNA sequence accession number and sequence length (bp)
3bIII	2011	MH diarrhea	++	1001	2-11-3-1-10-2-21 (ST167)	KM052166 990	KM112083 1286
4cI	2011	MH diarrhea	++	1001	2-11-3-1-10-2-21 (ST167)	KM052167 975	KM112082 1286
8dII	2011	MH diarrhea	++	1001	2-2-3-12-11-1-3 (ST8)	KM052168 1000	KM112081 1319
10cI	2011	mild diarrhea	++	1001	2-11-8-4-9-2-3 (ST168)	KM052169 971	KM112080 1299
21bI	2012	MH diarrhea	++	0001	2-13-3-6-10-2-3 (ST169)	KM052170 977	KM112079 1299
25cI	2012	MH diarrhea	++	1001	2-18-8-5-6-1-11 (ST170)	KM052171 1005	KM112078 1350
M1^a^	2011	MH diarrhea	++	1001	2-2-3-12-11-1-3 (ST8)	KM052172 933	KM112077 1175
M2^a^	2011	MH diarrhea	+	1001	2-2-3-12-11-1-3 (ST8)	KM052173 983	KM112076 1175
D1	2010	MH diarrhea	++	1001	2-11-8-5-10-2-6 (ST171)	KM052174 986	KM112075 1300
D28	2011	mild diarrhea	±	0001	2-11-3-20-6-2-21 (ST172)	KM052175 1005	KM112074 1182
B204 (ATCC 31212)	**–**	MH diarrhea	++	1001	1-16-3-4-2-3-6 (ST54)	U19610.1 1705	U14932.1 1433

^a^ Strains M1 and M2 were isolated on one farm, all other isolates originated from different, non-related farms.

^b^ Clinical signs on the farm from which each strain was isolated, MH = mucohemorrhagic.

^c^ ++ = strong, + = moderate, ± = weak.

^d^ indole production, hippurate hydrolysis, α-galactosidase, β-glucosidase (1 present, 0 absent).

^e^ allele numbers for adh-alp-est-gdh-glpK-pgm-thi. Between brackets: sequence type as assigned by Pub MLST database.

### Sequencing of 16S rRNA, NADH oxidase and Multi Locus Sequence Typing genes

The NADH oxidase (nox) gene and 16S rRNA gene were partially sequenced as previously described [39, 40]. The sequences retrieved from the isolates used in this study were compared by BLAST analysis to known sequences of *B.hyodysenteriae* type- and reference strains [41].

Multilocus sequence typing (MLST) of the *B.hyodysenteriae* isolates was based on the MLST scheme as previously published [42] and performed with modifications [43]. For all strains, sequences for genes encoding alcohol dehydrogenase (*adh*), alkaline phosphatase (*alp*), esterase (*est*), glutamate dehydrogenase (*gdh*), glucose kinase (*glpK*), phosphoglucomutase (*pgm*) and thiolase (*thi*) were determined and matched with the online MLST database [44]. The concatenated sequences of the described isolates, a previously typed Belgian strain Be45 [45], reference strains B204 (ATCC 31212) and WA1 (ATCC 49526) and four *B.* *intermedia* strains were aligned using ClustalW. The *B. intermedia* strains included the type strain PWS/A (ATCC 51140), and three strains isolated from pigs previously described by Råsbäck et al. [42]. A dendrogram was constructed using Kimura distance calculation and unweighed pair group method with averages (UPGMA).

### In vitro hemolysis assay

The hemolysis assay was based on the assays described by Fedorka-Cray et al. [46] and ter Huurne et al. [17] with some modifications. Fresh blood was collected from 8 week old pigs and a volume of blood was immediately mixed with an equal volume of Alsever’s solution (Sigma-Aldrich) (50/50 v/v). This blood-Alsever’s mixture was washed three times with Dextrose-Glucose-Veronal (DGV) (Lonza, Walkersville, MD, USA) buffered solution by centrifugation for 10 min at 500 *g*. The hematocrit of the suspension was determined using a micro-hematocrit centrifuge and reader. DGV buffered solution was added until a 10%-suspension of red blood cells was obtained.

Fresh cultures of the different *B.hyodysenteriae* isolates were prepared by harvesting a 4-day old culture plate with a sterile cotton swab and stirring the cotton swab in an anaerobic Brain Heart Infusion (BHI) broth (Bio-Rad), supplemented with 10% of Fetal Bovine Serum (FBS) (Thermo Fisher Scientific). These cultures were incubated for 24 h under anaerobic conditions at 37 °C on a rocking platform and for each strain three cultures were made. After incubation, cultures were microscopically examined for purity and the Optical Density at 620 nm (OD_620_) was measured. Cultures were only retained if their OD_620_ measured between 0.30 and 0.35. Supernatant was collected by centrifugation at 500* g* for 20 min and was sterilized by filtration (Millipore, 0.2 µm). The strongly hemolytic strain B204 (ATCC 31212) served as a reference strain in the in vitro hemolysis assay.

The hemolysis assay was performed in 96-well U-bottom microtiter plates. After pipetting 100 µL of the 10% red blood cell suspension in each well, 100 µL of the filtered *B.hyodysenteriae* culture supernatant was added. Triton-X 2% served as a positive control (complete hemolysis) and DGV served as a negative control (no hemolysis). Plates were incubated for two hours at 37 °C in a 5% CO_2_ atmosphere after which the 96-well plate was centrifuged for 10 min at 500 *g*. The supernatant of the incubated fluid was transferred to a 96-well IWAKI-plate and the absorption at 450 nm was determined using an ELISA-reader. All assays were performed in triplicate and repeated three times.

### Sequencing of hemolysis associated genes

Complete sequences of the *hlyA*, *tlyA*, *tlyB*, *tlyC*, *hemolysin III (BHWA1_RS02195)*, *hemolysin activation protein (BHWA1_RS02885)*, and *hemolysin III channel protein (BHWA1_RS09085)* genes were determined for all *B.* *hyodysenteriae* strains. For *hlyA*, the ACP1-Fo and ACP1-Re primers were used as described by Barth et al. [26]. For *tlyA*, the primers were designed based on the sequences of *tly* (GenBank: X61684.1) (*tlyA* was originally named *tly* as it was presumed to be the only hemolysin of *Brachyspira*) as deposited by Muir et al. [16] and the whole genome sequence of *B.hyodysenteriae* WA1 (GenBank: NC_012225.1) [24]. The *tlyB* and *tlyC* primers were based on the sequences (GenBank: X73140.1) (*tlyB*), (GenBank: X73141.1) (*tlyC*) [17] and their alignment with the whole genome sequence of WA1 respectively. Primers for *hemolysin III*, *hemolysin activation protein* and *hemolysin III channel protein* genes were designed based on the whole genome sequence of *B. hyodysenteriae* strain WA1 (GenBank: NC_012225.1) [26]. Primers, position as given in the whole genome sequence of WA1 (GenBank: NC_012225.1), product length and annealing temperature are shown in Table 2.

**Table 2:** **Primers, position, product size and annealing conditions for detection of hemolysis related genes** ***tlyA***, ***tlyB***, ***tlyC***, ***hemolysin III***, ***hemolysin activation protein*** **and** ***hemolysin III channel protein***

Target gene: primer names	Nucleotide sequence (5′ → 3′)	Position (NC_012225.1)	Product size (bp)	Temperature annealing (°C)
*tlyA: hemolysin A*				
tlyAS1Fo	GGTATTGGAGATGAATATAC	267 034–267 054	956	58
tlyAS1Re	TGATGTAGAAGGCTTCTATA	267 969–267 989		
*tlyB*: *hemolysin B*				
tlyBS3Fo	GGAGTGGAGAGAAAGTATTA	1 414 613–1 414 633	974	57
tlyBS3Re	TGCTGTAAGCAGACTTATAG	1 415 566–1 415 586		
tlyBS4Fo	AGCTGTCCTTCTTCAAGTAC	1 415 413–1 415 433	390	63
tlyBS4Re	AGTCGTAGGACAGAAAGAAG	1 415 782–1 415 802		
tlyBS2Fo	CCCTCTTCATAACCAACATA	1 415 533–1 415 553	1062	65
tlyBS2Re	AGGGACTTGCTGAAAAGATA	1 416 653–1 416 673		
tlyBS1Fo	TTGTACCAGCAACAACTGAA	1 416 575–1 416 595	1082	54
tlyBS1Re	AGCTCTATCTACAGCAATAC	1 417 635–1 417 655		
*tlyC*: *hemolysin C*				
tlyCFo	TTACGAATGCCTGCTATTTG	1 644 915–1 644 935	1131	50
tlyCRe	CTATTTTTAGGCGAGGCTTT	1 646 025–1 646 045		
*BHWA1_RS02195: hemolysin III*				
HlysCBSFo	GGAAAAAGGGATCCTGGAAC	704 725–704 745	1570	54
HlysCBSRe	TCCTGCTTGTTATCAGCACA	706 278–706 298		
*BHWA1_RS02885: hemolysin activation protein*				
Hlys3-1Fo	CTATTGGAGAGCGTACATCT	503 577–503 597	1014	58
Hlys3-1Re	TACCCTGTACCTACAGAACA	504 571–504 591		
*BHWA1_RS09085: hemolysin III channel protein*				
Hlys3-2Fo	CTCCTCCCGTTCAATATGTA	2 156 200–2 156 220	974	58
Hlys3-2Re	AATCCGCCATGTAAAACTGC	2 157 154–2 157 174		

PCR was performed under standard conditions in a 25 µL reaction volume with *Taq* polymerase (Bioline, Taunton, USA). The PCR program started with 95 °C for 15 min, followed by 35 cycles of 95 °C for 30 s, 1 min at the primer specific annealing temperature and 72 °C for 1 min. The final extension step was 72 °C for 2 min after which samples were cooled to 4 °C. Optimal annealing temperatures are given for each primer pair in Table 2. For all strains, the sequences were compared to each other and to the whole genome sequence of *B. hyodysenteriae* strain WA1 [26]. Furthermore, all sequences were compared to the whole genome sequences of 18 additional *B. hyodysenteriae* strains, including type strain B78 and reference strains B204 and FM88.90. These whole genome sequences were recently described by Black et al. [47].

### Statistical analysis

The in vitro hemolysis test results were analyzed by a one-way ANOVA, with Bonferroni corrections. A *P* value of <0.05 was considered significant and all statistical analysis was performed with the SPSS Statistics 22.0 software (SPSS Inc., Chicago, USA).

## Results

### Phenotypic and molecular identification of *B.* *hyodysenteriae* isolates

A collection of 35 *B.hyodysenteriae*, 15 *B.intermedia*, 7 *B.pilosicoli*, 12 *B.murdochii*, 10 *B.innocens* isolates, and 1 *B.hampsonii* isolate has been assembled. During the characterization of the strain collection it was noted that one isolate, M2, that was donated by a diagnostic laboratory, showed only moderate hemolysis on TSA plates supplemented with 5% sheep blood, although it had been presented as a *B.hyodysenteriae* isolate. Another isolate, D28, had been presented as *B. murdochii* by a diagnostic laboratory. This isolate was phenotypically identifiable as *B.* *murdochii*, but was positive in all *B.* *hyodysenteriae* specific PCR’s and negative in all species-specific PCR’s for other *Brachyspira* sp..

For the final selection of ten isolates, results of the phenotypic characterization are shown in Table 1. Most isolates showed strong hemolysis after growth for four days on TSA plates supplemented with 5% sheep blood. However, as mentioned previously, isolate M2 showed only moderate hemolysis and isolate D28 showed weak hemolysis. Eight out of ten isolates were indole positive and two were negative.

The ten selected strains tested positive in the *B.hyodysenteriae* specific PCRs based on 23S rRNA, *nox* and *tlyA* genes. Sequences of the *nox* genes of all the isolates showed 100% similarity to previously described *B.* *hyodysenteriae* strains retrieved from GenBank. For the ten selected strains the *nox* gene sequences were identical, except for strain 25cI. The 16S rRNA gene sequence of these strains also showed 100% similarity to previously described *B.hyodysenteriae* strains retrieved from GenBank. All sequences were deposited in GenBank, accession numbers and sequence length are given in Table 1.

**Figure 1:**
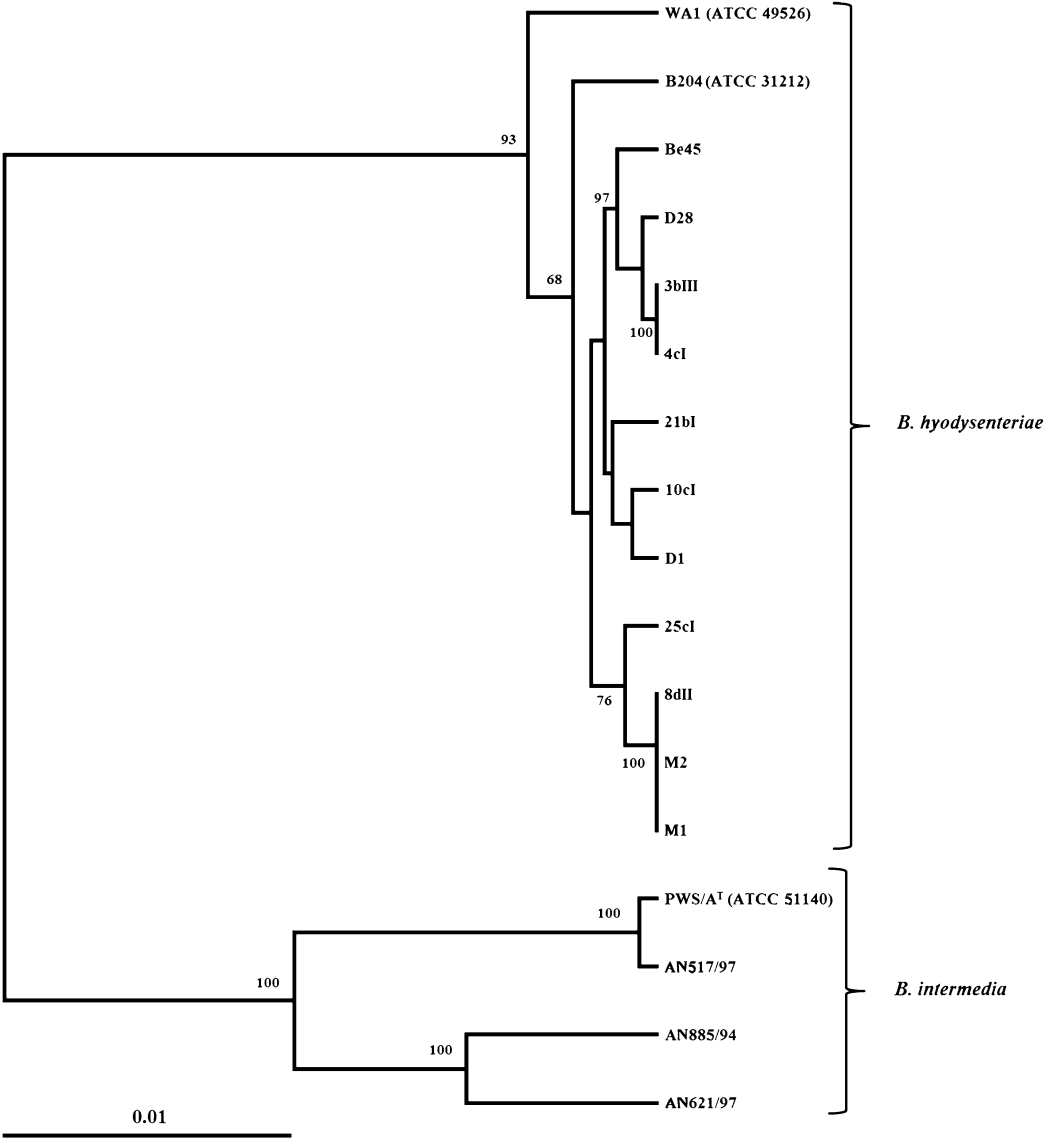
**Dendrogram based on the concatenated sequence (4086** **bp) of the 7 MLST genes of** ***B. hyodysenteriae*** **and** ***B.*** ***intermedia.*** The alignment was created using clustalw, for the dendrogram distance calculation (Kimura) and UPGMA were used (PHYLIP). Bootstrap values greater than 60 are shown in the nodes. Scale bar indicates a distance of 1 substitution in 100 nt.

MLST results are given in Table 1. All 7 genes could be amplified and sequenced for the described isolates. The MLST profiles of isolates 8dII, M1 and M2 are identical and have previously been deposited as sequence type 8. All other profiles represent new sequence types but have four or five loci in common with already existing profiles in the pubMLST database. A dendrogram based on the concatenated sequence (4086 bp) of the 7 MLST genes of *B.hyodysenteriae* and *B.intermedia* is given in Figure 1.

### In vitro hemolysis of *B.hyodysenteriae* strains shows gradual variation

**Figure 2:**
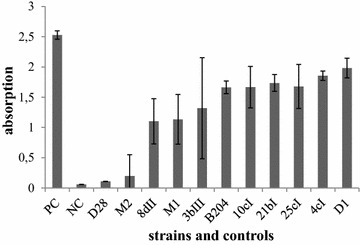
**In vitro** **hemolytic capacity of** ***B. hyodysenteriae*** **strains used in this study.** Hemolysis is represented by the mean value of absorption at 450 nm after incubation of red blood cell suspension with supernatant of the different *B. hyodysenteriae* identifiable strains. PC, positive control; NC, negative control. Significant differences between *B. hyodysenteriae* identifiable strains and reference strain B204 are indicated,* *P* < 0.01.

Figure 2 displays the in vitro hemolysis of the described *B.hyodysenteriae* strains. The strength of hemolysis showed gradual variation, nevertheless most strains showed a strength of hemolysis in the same range as the B204 reference strain. For strain D28 and M2 the hemolysis was significantly lower than for the B204 reference strain (*P* < 0.01).

### Nucleic acid and amino acid substitutions in hemolysis associated genes

The sequences for *hlyA* were identical to the whole genome sequences of WA1 and the 18 additional strains [47], except strain 3bIII and 4cI, which differed with regard to two nucleotides. However, these nucleotide differences were synonymous and did not translate into a different amino acid sequence. The positive result for all strains in the *hlyA*-ACP PCR also showed that the *hlyA* gene was placed as expected between the accompanying *fab*-F and *fab*-G genes, coding for an ACP-reductase and –synthetase [25].

Weakly hemolytic strain D28 was the only strain with a nucleic acid substitution in the *tlyA* gene. The substitution was located at position 501 (G → T) as given in Tly (GenBank: X61684.1) by Muir et al. [16] or position 267228 as in the genome sequence of WA1 (GenBank: NC_012225.1) [24]. This non-synonymous nucleic acid substitution translated into a different amino acid at position 10 in the amino acid sequence (Glycine → Cysteine). In all other whole genome sequences the sequence of *tlyA* was identical to WA1, except for strain ST195 were there was a synonymous substitution in one nucleotide at position 938 (A → C) as given in Tly (GenBank: X61684.1) by Muir et al. [16] or position 267725 as in the genome sequence of WA1 (GenBank: NC_012225.1) [24].

The sequence of the *tlyB* gene showed differences between the isolates and the number of nucleotide changes varied from 1 to 7 as given in Table 3. For all strains, except the weakly hemolytic strain D28, these nucleotide differences were synonymous. The sequence of strain D28 differed at two positions of which the nucleotide change at position 1416206 (C → T) translates into an amino acid substitution at position 384 in the amino acid sequence (Alanine → Threonine). In all other whole genome sequences only one strain (ST195) was reported to have a synonymous substitution [47].

**Table 3:** **Nucleotide and amino acid differences for hemolysis related genes of** ***B.*** ***hyodysenteriae*** **identifiable** **strains used in this study**

Strain	In vitro hemo-lysis	*tlyA* 723 nt	*hlyA* 237 nt	*tlyB* 2487 nt	*tlyC* 807 nt	*Hemolysin III* 1335 nt	*Hemolysin activation protein* 675 nt	*Hemolysin III channel protein* 672 nt
3bIII	++	0	2 (0)	7 (0)	0	0	15 (5)	2 (1)
4cI	++	0	2 (0)	7 (0)	0	0	15 (5)	2 (1)
8dII	++	0	0	0	0	10 (0)	15 (5)	0 (0)
10cI	++	0	0	5 (0)	0	0	14 (5)	1 (0)
21bI	++	0	0	1 (0)	0	0	15 (5)	1 (0)
25cI	++	0	0	7 (0)	0	0	14 (5)	1 (0)
D1	++	0	0	5 (0)	0	0	14 (5)	1 (0)
D28	±	1 (1)	0	2 (1)	4 (0)	63 (5)	44 (8)	12 (1)
M1	++	0	0	0	0	10 (0)	15 (5)	0 (0)
M2	+	0	0	0	0	10 (0)	15 (5)	0 (0)

Differences compared with the genome sequence of *B.* *hyodysenteriae* strain WA1. Number of amino acid changes are given in brackets.

With regard to the *tlyC* gene, all strains were identical to WA1 and all other whole genome sequences except for weakly hemolytic strain D28 of which the *tlyC* sequence differed in four nucleotides. Nonetheless this altered nucleotide sequence consisted of synonymous substitutions only.

The *hemolysin III* gene sequence (*BHWA1_RS02195*) showed no nucleotide differences for seven of the strains. The strains 8dII, M1 and M2 shared an identical sequence which diverged 10 nucleotides compared to the sequence of *B.hyodysenteriae* reference strain WA1. However, these nucleotide differences did not translate into a different amino acid sequence. The weakly hemolytic strain D28 showed 68 nucleotide differences compared to the sequence of *B.hyodysenteriae* reference strain WA1. These nucleotide differences resulted in 5 amino acid substitutions at following positions: 81 (Valine → Isoleucine), 113 (Methionine → Valine), 164 (Glutamic acid → Aspartic acid), 227 (Threonine → Serine), 264 (Valine → Isoleucine). The majority of the other whole genome sequences showed a *hemolysin III* gene sequence identical to WA1, 6 strains showed synonymous nucleotide substitutions and strain B6933 had two amino acid substitutions at position 241 (Methionine → Isoleucine) and 335 (Valine → Isoleucine).

With regard to the *hemolysin activation protein* gene (*BHWA1_RS02885*) all strains showed a difference of 14 or 15 nucleotides with the sequence of *B.* *hyodysenteriae* reference strain WA1 (Table 3). These sequences translated in five amino acid sequence differences at following positions: 51 (Proline → Serine), 56 (Valine → Isoleucine), 59 (Valine → Leucine), 82 (Leucine → Isoleucine), 93 (Valine → Isoleucine). Strain D28 showed 41 nucleotide differences compared to the sequence of *B.* *hyodysenteriae* reference strain WA1 (Table 3), which translates into an amino acid sequence different from that of strain WA1 by 8 amino acids: 47 (Threonine → Isoleucine), 49 (Valine → Methionine), 56 (Valine → Isoleucine), 79 (Valine → Isoleucine), 82 (Leucine → Isoleucine), 111 (Valine → Isoleucine), 114 (Leucine → Proline), 133 (Methionine → Isoleucine). The whole genome sequences of the 18 additional *B. hyodysenteriae* strains showed various amino acid substitutions compared to WA1. Six strains shared the five amino substitutions as seen in most of the strains of this study, strains B204, B6933 and B78 showed one additional amino acid substitution at position 157 (Lysine → Glutamic acid). One strain (NSW15) showed three amino acid substitutions compared to WA1 at positions 19 (Lysine → Arginine), 133 (Methionine → Isoleucine), 180 (Isoleucine → Methionine), and strains Q17, B8044 and 865 showed four amino acid substitutions compared to WA1 at positions 54 (Isoleucine → Methionine), 82 (Leucine → Isoleucine), 93 (Valine → Isoleucine) and 157 (Glutamic acid → Lysine).

The sequences for *hemolysin III channel protein* gene (BHWA1_RS09085) of the strains in this study were either identical to that of *B.* *hyodysenteriae* reference strain WA1, differed by 1 or 2 nucleotides, or differed by 12 (strain D28). For strains 3bIII and 4cI this resulted in an amino acid substitution at position 217 (Arginine → Isoleucine), and for strain D28 at position 209 (Valine → Isoleucine). For the other whole genome sequences seven strains showed an identical *hemolysin III channel protein* gene sequence to WA1, nine strains shared a synonymous nucleotide substitution at position 2156792 as given in the genome sequence of WA1. Strain B78 showed one amino acid substitution at position 120 (Alanine → Threonine).

Table 3 displays the number of nucleotide and amino acid differences for the sequences of the *hlyA*, *tlyA*, *tlyB*, *tlyC*, *hemolysin III*, *hemolysin activation protein* and *hemolysin III channel protein* genes between the *B.hyodysenteriae* strains in comparison with the genome sequence of *B.hyodysenteriae* reference strain WA1. All sequences have been deposited in GenBank (accession numbers KM112034-KM112073, KU215622-KU215658).

## Discussion

This study describes quantification of hemolytic capacity of *B.* *hyodysenteriae* strains, and provides evidence that the degree of hemolysis can vary within the species *B.hyodysenteriae.* The phenotypic characterization tests, species-specific PCR, and sequences of the *nox* and 16S rRNA genes of moderately or weakly hemolytic strains show that these strains belong to the species *B.hyodysenteriae.* The dendrogram based on the MLST results (Figure 1) shows that the weakly hemolytic *B.hyodysenteriae* strains are nested within clades containing strongly hemolytic *B.hyodysenteriae* strains. Even if only DNA/DNA hybridization might be considered sufficiently accurate enough to effectively identify a strain, the strains described here would undoubtedly be identified as *B.hyodysenteriae* in all currently used methods for genetic identification (PCR, *nox* and 16S rRNA sequencing, MLST).

The comparative sequence analysis of the hemolysis associated genes leads to a hypothesis with regard to the underlying mechanism of the weak hemolysis. The weakly hemolytic *B.hyodysenteriae* strain D28 possesses nucleotide sequence differences in the *tlyA*, *tlyB*, *hemolysin III*, *hemolysin activation protein* and *hemolysin III channel protein* genes resulting in amino acid substitutions. These sequences differ from those of all other strains in the study and from that of reference strain WA1. Whether the amino acid substitutions reported here are the sole reason for the weak hemolysis of this strain needs further studies. In our opinion the most important genes involved in the strong hemolytic phenotype of *B.hyodysenteriae* are *tlyA, hlyA* and probably *hemolysin III.* Deletion mutants for *tlyA* have been reported to be weakly hemolytic on blood containing agar plate [23]. The role of ACP in acylation of toxins has been demonstrated for other toxins, such as RTX toxins [24], which makes it likely that *hlyA* encoding an ACP plays a role in the hemolytic capacity of *B.hyodysenteriae.* Hemolysin III harbors a conservative domain yqfA, a predicted channel-forming protein of the hemolsyin III family, which might indicate its role in *B.hyodysenteriae* hemolysis. Whether this reduced hemolytic capacity can be attributed to one of the amino acid changes in one of the hemolysis associated genes, remains to be determined. In order to completely elucidate this, the construction of specific mutants of *B.hyodysenteriae* which harbor one of the divergent hemolysis associated genes is a prerequisite. This might be hampered by the fact that is difficult to genetically manipulate *B.hyodysenteriae.*

Not only a difference in amino acid sequence, which can affect the function of a protein, might influence the gradation in hemolytic capacity but there might also occur a more distant variance such as altered activity of promoter regions or altered transcription of genes under specific circumstances in vitro as well as in vivo. Although repeated subculturing can result in phenotypical changes such as loss of hemolysis [48], this has, to our knowledge, not been described for *B.hyodysenteriae.* Besides, already during primary isolation of strains D28 and M2, hemolysis was always weak and moderate, respectively.

*Brachyspira hyodysenteriae* strain M2 is only moderately hemolytic. However, the nucleotide sequence differences observed for strain M2 did not result in amino acid changes except for the *hemolysin activation protein* gene. However, for this gene amino acid substitutions were observed for all investigated strains compared to WA1. It should be mentioned that unlike D28, which originated from a farm where only mild diarrhea was present, M2 originated from a farm where pigs were suffering from mucohemorrhagic diarrhea. Alongside M2, another isolate M1, originated from the same farm. The presence of different strains with divergent biological properties on one farm could influence the outcome of control measurements, since these strains may differ in other biological properties as well, such as their antimicrobial resistance.

Strain D28 originated from a farm were only mild diarrhea was present. In preliminary trials, in which pigs were inoculated with this strain, no symptoms of SD were observed, even if the strain was shed in the feces of the inoculated pigs at 10^7^copies/g feces. Even though the significance of the presence of weakly hemolytic strains of *B.hyodysenteriae* in a herd as a hazard for porcine health is not clear at the moment, the mere occurrence of weakly hemolytic strains of *B.hyodysenteriae* poses problems for the diagnosis of swine dysentery. When diagnosis is primarily based on microbial culture procedures, these strains could be mistaken for *B.intermedia* or *B.murdochii*, since the phenotypic profile of weakly haemolytic, indole positive *B.hyodysenteriae* is equal to that of *B.intermedia* and the phenotypic profile of weakly haemolytic, indole negative *B.hyodysenteriae* is equal to that of *B.murdochii.* When diagnosis is primarily based on the current PCR tests, the degree of hemolysis of the specific strain cannot be estimated. If a herd tests positive for *B.hyodysenteriae*, this may influence the trading possibilities of the farm in question, because of the possible risk of *B.hyodysenteriae* carrier animals. In order to avoid misdiagnosis, the combination of phenotypic characterization and PCR, complemented with sequencing if necessary, is presumably the most complete method for species identification of *Brachyspira* sp. for now.

Although in our collection of 35 isolates, spanning a time-period of 2 years, only two *B.hyodysenteriae* strains were found with an aberrant hemolytic phenotype, appearance of weakly hemolytic, possibly low virulent strains of *B.hyodysenteriae* may affect herd dysentery status, with great impact on a farms trading opportunities. The prevalence of weakly hemolytic *B.hyodysenteriae* could be underestimated since it has not been regularly looked for or could go unnoticed if PCR and microbial culture are not combined.
